# Socio-demographic factors associated with health conditions and treatment-seeking choices in border communities of Ogun state Nigeria: a cross-sectional study

**DOI:** 10.3389/fpubh.2026.1914604

**Published:** 2026-09-25

**Authors:** Henry Osita Chukwu, Dominic Azuh, Muyiwa Oladosun

**Affiliations:** Department of Economics and Development Studies, Demography and Social Statistics, Covenant University Ota, Ota, Nigeria

**Keywords:** health, Nigeria, Ogun state, residents in border communities, socio-demographic factors

## Abstract

**Background:**

Border communities in Nigeria are mostly neglected and marginalized rural and remote areas. Hence they face unique health challenges due to scarcity of basic amenities and infrastructure such as clean water, schools, adequate healthcare facilities and services among others. Data on health conditions and treatment-seeking choices in these border areas are limited. Understanding socio-demographic factors associated with health conditions and treatment-seeking choices is vital for proper intervention. This study examined these associations in Ipokia, Yewa North and Imeko-Afon LGAs of Ogun State Nigeria.

**Methods:**

The study conducted a community-based cross-sectional study with 1,317 respondents aged 18 years and above, from 16th September 2024 to 22nd November 2024, in selected border communities within these three LGAs. A multi-stage sampling technique was used to select these respondents. Structured interviewer-administered questionnaire were used to collect data from the respondents. The socio-demographic characteristics of the respondents were used as the independent variables, while health condition and treatment-seeking choices denoted the dependent variables. Univariate and binary logistic regression analyses were performed on the data using SPSS version 20.

**Results:**

Results show that socio-demographic factors such as age, location, duration in location, level of education, income level and occupation of respondents are significantly associated with the health conditions and treatment-seeking choices of residents in border communities of Ogun State, Nigeria. The study recommends provision of adequate health services, more investments in education and economic empowerment in these border areas, which will help to improve their health outcomes.

## 1 Background

By definition, socio-demographic factors are the social and demographic characteristics of individuals or population, such as age, sex, marital status, level of education, income level among others. For this study, these comprise age, sex, marital status, number of children, location of respondent, duration in location (number of years living in location), level of education, income level, occupation and religion. These factors may influence exposure to health risk, access to healthcare, health literacy and ability to make health related choices. Health condition in this context refers to the respondent’s self-reported state of health within the last 12 months before the study. And treatment-seeking choices as used in this study imply the different methods adopted by the respondents in procuring cure for their stated illnesses. Ogun State in Nigeria shares borders with the Benin Republic. Given the disparities in the socio-demographic characteristics of people living in border regions in Nigeria, compared to other parts of the country, it is important to investigate the effects of socio-demographic factors on the health of residents of border communities in Nigeria. Border settlements in Ogun State are mostly neglected and marginalized rural and remote areas, perhaps because they lie at the periphery of the country; far away from the center where economic and social infrastructural developments are concentrated. Hence these border areas lack necessary economic empowerment, and basic social amenities such as schools, adequate health services, access to clean water, among others, which are vital for maintaining good health. This situation contributes in the socio-demographic composition, and general wellbeing of residents of these communities. Due to peripheral locations, distance from central health infrastructure, and high out-of-pocket costs, residents often delay seeking treatment or adopt other measures, which exacerbate clinical outcomes. As a result, morbidity trends and treatment-seeking choices in these communities are influenced not only by individual characteristics, but also by the quality of policies and interventions targeted at these communities, by relevant authorities. Addressing these regional health disparities requires focused public health education and improved social amenities. This study examines the connection between socio-demographic factors, health conditions and treatment-seeking choices in border communities of Ogun State Nigeria, so as to ascertain the influence that these socio-demographic variables have on the health of people who live in these localities. Good health is important as it enhances productivity in labor, growth of the nation and general wellbeing of the people (1).

Theoretically, according to the Anderson Model, socio-demographic factors such as age, sex, and education are predisposing factors which influence an individual’s propensity to become sick and their likelihood of using health services. Enabling factors such as income, availability of, and distance to health facilities shape formal treatment or alternative care choices. Need factors include perceived and evaluated health status, and determines whether the individual seeks care or not (2). In addition, the Health Belief Model imply that education and income shape perception of disease severity and barriers to treatment-seeking, hence affecting care choices (3). Thus lower education and lower income will be significantly associated with higher morbidity, and preference for informal treatment-seeking choices. Moreover, the Social Determinants of Health, posits that structural determinants such as education, income and occupation, plus other intervening influences like healthcare access and living conditions shape health outcomes (4). These inequalities in health spring up due to the circumstances in which people live, work, and age, plus the prevailing health system in existence (4). Hence, in Ogun State border communities, enabling factors such as availability and cost of healthcare services, plus distance to health facilities could influence treatment-seeking choices, leading to preference for available, and informal cheaper alternatives, like traditional medicine and self-medication.

Braveman & Gottlieb (5) argued that the frequency and distribution of both infectious and chronic diseases are directly affected by the social determinants of health. Social determinants of health include the conditions or circumstances in which people are given birth to, grow, work and age. These circumstances are influenced by the historical and current distribution of money, power and resources; at local, regional and global stages. Moreover, these determinants depend directly on the policies implemented at these levels, and also on the health system, making them persistent, systemic and preventable (6). In comparison to people in higher social classes, individuals in lower social strata are two times more likely to experience serious sicknesses and death (7). These inequalities emanate from the socio-economic and socio-political situations in which people live, hence exposing them to peculiar risk circumstances (5).

Social disparities shape social determinants of health and impact health conditions, access to services, and trend of morbidity and mortality among people (8). Moreover, an individual’s area of residence influences access to public goods. For instance, in rural areas, there may be greater difficulties in accessing healthcare, education and public transport (9). Fantin et al. (10) implied that communities with low economic status usually experience significant difficulties accessing basic health services. People who have greater economic power and access to resources are more likely to be better off, when compared to those in deprived positions, resulting in inequalities in health (11). The high rate of poverty in Nigeria requires adequate response from government and policy makers, so as to checkmate the challenge (12). In Africa, the availability of social protection contributes to reduction in inequality and poverty (13).

Moreover, Tarar et al. (14) examined the influence of socio-demographic factors on parental health-seeking behavior for illnesses among the pediatric age group in Karachi. The authors found out that most of the parents (82.7%) sought healthcare for their children from medical doctors, followed by spiritual healers (10.7%), traditional healers (5.6%) and homeopathic doctors (0.9%). Mou et al. (15) examined the influence of socio-demographic factors on health-seeking behaviors among residents in Bangladesh during the first wave of COVID-19. The authors stated that higher health-seeking behaviors were reported among people of older age (greater than 50 years), higher education, higher income, and educated unemployed students. Whereas, people who live in slums, and who do business as source of income showed lower health-seeking behavior. Also, females exhibited better health-seeking behaviors compared to males. Nachinaab et al. (16) reiterated that there were significant relationships between socio-demographic factors such as marital status, sex, income level and education, and the health-seeking behavior of patients with respect to the utilization of private and public health facilities.

Ogbonna et al. (17) explored socio-demographic risk factors for mental health disorders among school children in Umuahia, South East Nigeria. The authors stated that children within the age group of 10–13 years were 8 times more likely to have mental health disorders than children in the other age categories. Also, female children were 3 times more likely to develop mental health disorders, compared to male children. Additionally, children of the lower socio-economic status were 3 times more likely to have mental health disorders than children from other socio-economic status category. Moreover, children who reside with widowed caregivers were 5 times more likely to develop mental health disorders than children who stay with caregivers that belong to another marital status. In addition, children who reside with non-biological caregivers were 4 times more likely to have mental health disorders, compared to those who live with biological caregivers.

Furthermore, Braimah et al. (18) estimated demographic and socio-economic factors that drive dissatisfaction with the formal utilization of healthcare services among very low income older adults in Ghana. The paper stated that females and people with high school education were less likely to show dissatisfaction with the utilization of formal healthcare services than males and people with no formal education. Also, the study showed that individuals who earned a monthly income of 201 Ghana cedi or more were more likely to claim dissatisfaction when utilizing formal healthcare services compared to those who earned less than 100 Ghana cedi per month.

According to Chukwu et al. (19, 20), border residents with poorer access to potable water experience higher self-reported illness rates. Women engaged in informal cross-border trade along the Nigeria-Benin border face numerous problems which include gender-based violence and sexual exploitation from border control agents (21), and this has resulted in a public health challenge (22). The health system in Nigeria is among the weakest in the world, and it is characterized by the concentration of nurses and doctors in a few tertiary health facilities in cities, whereas, rural areas that make up more than 70% of the population suffer from a shortage of health personnel and insufficient healthcare facilities (23).

## 2 Data and methods

### 2.1 Research design

This study was conducted in Ogun State, Nigeria. Ipokia, Yewa North and Imeko-Afon Local Government Areas (LGAs), which are the only three LGAs in Ogun State, Nigeria that share borders with the Benin Republic (24), as at the time of conducting this research were the locations used for the study. The study was conducted using structured questionnaires. These questionnaires were administered to selected households in each of the border communities visited. The researcher adopted content validity for this study by making sure that all the questions asked in the questionnaire included all that are implied by the research questions. The study also used a panel of judges to review the questions in the questionnaire carefully, considering their relevance to the subject matter and their comprehensive coverage of the entire topic of study. The study adopted a multi-stage sampling technique. The three LGAs that share borders with the Benin Republic were first identified. Selected communities within these LGAs were purposively chosen for the study, followed by selection of enumeration areas within the communities. Then there was a systematic random selection of the households from where the respondents were sampled.

Moreover, in every household visited, the interviewer interviewed any member of the household who was 18 years or above (legal consent age in Nigeria). Using the Taro Yamane formula to calculate the sample size as 1,317 respondents, a proportional sample of 474 respondents were interviewed in the Ipokia LGA, 580 respondents were interviewed in the Yewa North LGA and 263 respondents were interviewed in the Imeko-Afon LGA. The data collection for the study was done by the researcher and eight research assistants. In recruiting the research assistants, the researcher ensured that the research assistants could fluently speak the local dialect being used in the study location, in addition to fluency in English. The research assistants were specifically trained using the questionnaire for the study, prior to being taken to the field for the proper study. The researcher keenly monitored and supervised the data collection process in order to mitigate interviewer’s bias or influence. The study was carried out from 16th September 2024 to 22nd November 2024 (Figures 1, 2).

**Figure 1:**
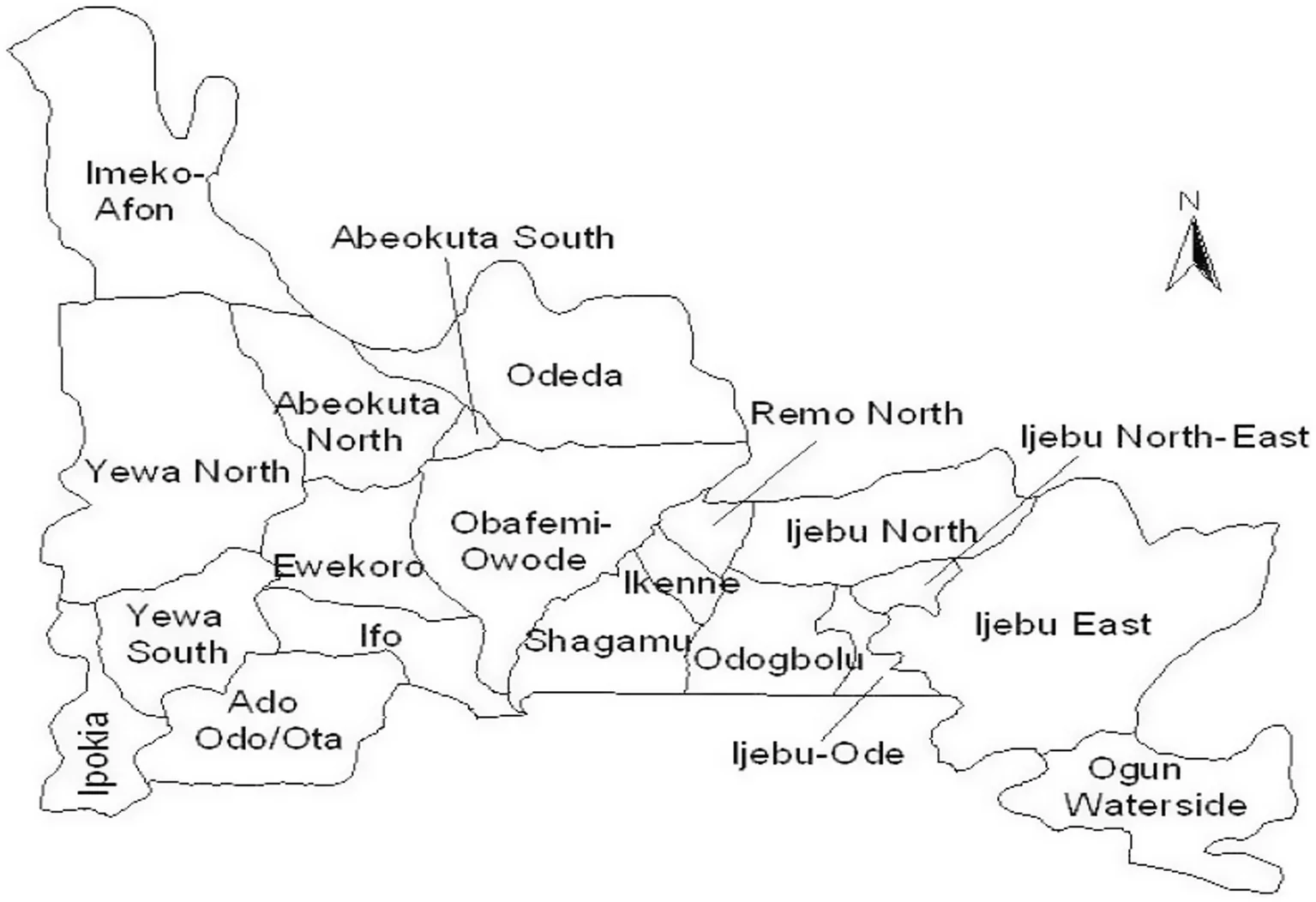
Map of Ogun State, showing its LGAs. Source: Oladehinde et al. (24).

**Figure 2:**
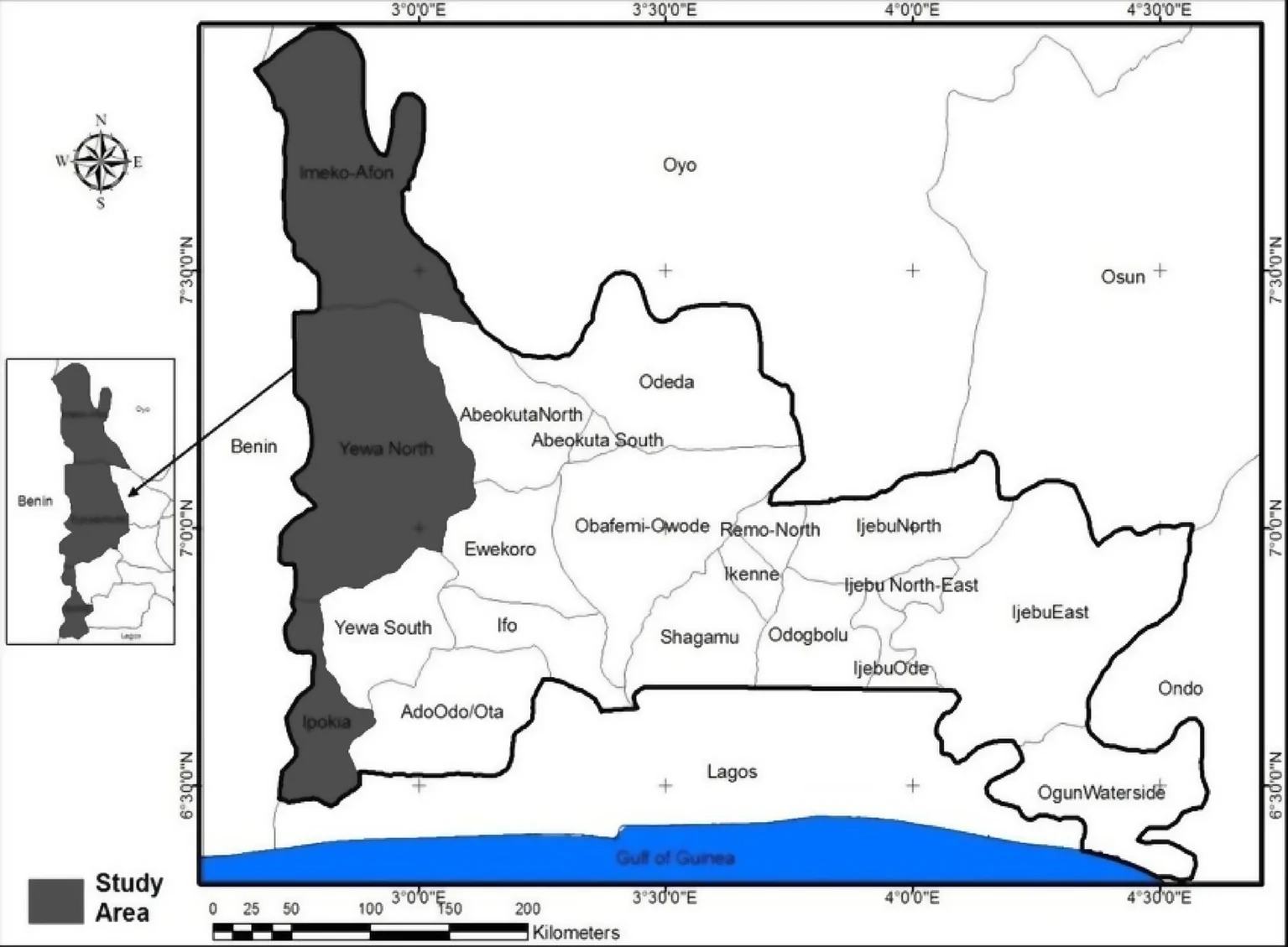
Map of Ogun State showing the study area. Source: Oladehinde et al. (24).

### 2.2 Model specification

The dependent variables (Y) for this research are the health condition and treatment-seeking choices of the respondents, which include (1) sick in the last 12 months before the study (2) frequency of sickness (3) cure through hospital treatment (4) cure through traditional medicine and (5) cure through self-medication. The independent variables (X) are the socio-demographic characteristics of the respondents. The dependent variable is said to be a function of the independent variables (X). The Model Specification is as shown in Equations 1–5:

The relationship is represented as;

Y=f(X) (1)

$$Y=f(\begin{array}{l} \mathrm{AOR},\mathrm{SOR},\mathrm{MSR},\mathrm{NCR},\mathrm{LOR},\\ \mathrm{DIL},\mathrm{LOE},\mathrm{ILR},\mathrm{OCR},\mathrm{ROR} \end{array})$$ (2)

Where;

Independent variables:

AOR = Age of respondent.

SOR = Sex of respondent.

MSR = Marital status of respondent.

NCR = Number of children of respondent.

LOR = Location of respondent.

DIL = Duration in location by respondent.

LOE = Level of education of respondent.

ILR = Income level of respondent.

OCR = Occupation of respondent.

ROR = Religion of respondent.

The estimation technique employed to estimate the model used in this study is Binary Logistic Regression technique;

$$Y=\beta_{o}+\beta_{1}x_{1}+\beta_{2}x_{2}+\ldots +\beta_{n}x_{n}+e_{t}.$$ (3)

Where;

Y = dependent variable.

β _o =_ intercept.

β _1_ −β _n_ = the coefficients of the variables.

x _1_ –x _n_ = the independent variables.

e_t_ = the error term.

The logistic model is represented by the regression equation below;

$$Y=\log(\frac{p}{1-p})=\beta_{o}+\beta_{1}x_{1}+\beta_{2}x_{2}+\ldots {+}_{n}x_{n}+e_{t}.$$ (4)

$$\begin{array}{l} Y=\beta_{o}+\beta_{1}\mathrm{AOR}+\beta_{2}\mathrm{SOR}+\beta_{3}\mathrm{MSR}\\ +\beta_{4}\mathrm{NCR}+\beta_{5}\mathrm{LOR}+\beta_{6}\mathrm{DIL}+\beta_{7}\mathrm{LOE}\\ +\beta_{8}\mathrm{ILR}+\beta_{9}\mathrm{OCR}+\beta_{10}\mathrm{ROR}+e_{t}. \end{array}$$ (5)

### 2.3 Data analysis

The data analyses focused on the responses of respondents in the three stated LGAs. Using SPSS version 20, the study conducted multivariate analysis to examine the degree of relationship between the independent variable(s) and the dependent variable(s). The socio-demographic factors were the independent variables. For the dependent variables, health condition of the respondent was measured by (1) whether the respondent was sick in the last 12 months and (2) frequency of sickness. While treatment-seeking choice was measured by (3) whether the respondent got cure through hospital treatment (4) whether the respondent got cure through traditional medicine and (5) whether the respondent did self-medication. It is also important to note that 855 respondents reported presence of sickness in these border communities during this study. Multicollinearity was assessed using the variance inflation factor (VIF), and all values were below 3, signifying that multicollinearity does not pose a serious concern in this study (25).

### 2.4 Ethical considerations

The study primarily employed a primary data collection method using structured questionnaires administered to respondents; hence there was a one-on-one interaction with the respondents. The researcher took utmost care to ensure that the privacy rights and personal concerns of the respondents were not violated. To achieve this, the researcher obtained ethical approval for this study from the Covenant Health Research Ethics Committee (CHREC), with the HREC Protocol Assigned Number CHREC/420/2023. The study adhered strictly to the rules of ethics for investigation of human subjects as laid down by CHREC; participants were duly informed of the purpose and nature of the study, and that participation was entirely voluntary, as each participant could withdraw at any stage in the discussion. The researcher also assured the respondents of the anonymity of the information they provide. Everyone who participated in the survey accepted a written informed consent before the process proceeded.

## 3 Results

### 3.1 Socio-demographic profile of the respondents

Table 1 presents the socio-demographic profile of the respondents. Table 1 shows that 14.0% of the respondents were aged 18–27 years, 24.9% were within ages 28–37 years, 31.3% were in the age group of 38–47 years, 18.8% were aged 48–57 years, 8.2% fall within ages 58–67 years, 2.5% were aged 68–77 years, while 0.3% were 78 years old and above. Also, 54.4% of the respondents were females and 45.6% were males. Furthermore, 14.0% of the respondents were single, 74.2% were currently married, 5.3% were divorced/separated and 6.5% of the respondents were widowed. Moreover, 8.9% of the respondents have one child, 27.3% have two children, 26.0% have three children, 16.3% have four children, and 5.5% have five children, while 16.0% fall within the ‘other’ category. In addition, 36.0% of the respondents were interviewed in Ipokia LGA, 44.0% in Yewa North LGA and 20.0% in Imeko-Afon LGA. Furthermore, 7.7% of the respondents have lived in the border communities for 1–10 years, 9.3% have lived for 11–20 years, 23.4% have dwelled in the border communities for 21–30 years, 25.7% have stayed for 31–40 years, 19.4% have lived for 41–50 years, 11.4% have dwelled in these border communities for 51–60 years, and 3.2% have stayed for 61 years and above. Moreover, 9.6% of the respondents have no formal education, 28.2% of the respondents have primary education, 51.3% have secondary education, 3.0% have post-secondary education and 7.8% were tertiary educated. For income level, 49.1% of the respondents earn less than 30,000 naira per month, 33.4% earn between 30,000 naira and 69,999 naira per month, 12.8% earn between 70,000 naira and 99,999 naira per month, 4.6% earn between 100,000 naira and 129,999 naira per month, while 0.1% fall within the ‘other’ category. Concerning occupation, 5.7% of the respondents were not working, 21.1% were self-employed, 2.7% were civil servants, 11.0% were into farming, 34.3% of the respondents were into trading, 20.3% were artisans and 4.9% fell under the ‘other’ category. Also, 56.3% of the respondents were Christians, 38.3% practiced Islam, 5.2% were Traditionalists, and 0.1% fell under the ‘other’ category.

**Table 1:** Respondents’ socio-demographic characteristics.

Variables	Freq.	Percent
Socio-demographic factors		
Age (Years)		
18–27	184	14.0
28–37	328	24.9
38–47	413	31.3
48–57	247	18.8
58–67	108	8.2
68–77	33	2.5
78 and above	4	0.3
Sex		
Female	717	54.4
Male	600	45.6
Marital status		
Single	184	14.0
Married	977	74.2
Divorced/Separated	70	5.3
Widowed	86	6.5
Number of children		
1	117	8.9
2	359	27.3
3	342	26.0
4	215	16.3
5	73	5.5
Other	211	16.0
Location of respondent		
Ipokia LGA	474	36.0
Yewa North LGA	580	44.0
Imeko-Afon LGA	263	20.0
Duration in location (Years)		
1–10	102	7.7
11–20	122	9.3
21–30	308	23.4
31–40	338	25.7
41–50	255	19.4
51–60	150	11.4
61 and above	42	3.2
Level of education		
No formal education	127	9.6
Primary education	372	28.2
Secondary education	676	51.3
Post Secondary education	39	3.0
Tertiary education	103	7.8
Income level (Naira/Month)		
Less than N30,000	647	49.1
N30,000–N69,999	440	33.4
N70,000–N99,999	169	12.8
N100,000–N129,999	60	4.6
Other	1	0.1
Occupation		
Not working	75	5.7
Self-employed	278	21.1
Civil servant	36	2.7
Farming	145	11.0
Trading	452	34.3
Artisan	267	20.3
Other	64	4.9
Religion		
Christian	742	56.3
Islam	505	38.4
Traditionalist	69	5.2
Other	1	0.1
N	1,317	100

### 3.2 Binary logistic regression showing the effects of socio-demographic factors on health condition and treatment-seeking choices

Table 2 presents the relationships between respondents’ socio-demographic factors, health condition and treatment-seeking choices among residents in these border communities based on logistic regression models.

**Table 2:** Binary logistic regression on health condition and treatment-seeking choices by socio-demographic factors.

Socio-demographic factors	Model 1		Model 2	
	Sick in the last 12 months		Frequency of sickness	
	OR (95% C. I)	Sig.	OR (95% C. I)	Sig.
Age (Years)				
18–27	1.000		1.000	
28–37	0.724(0.459, 1.142)	0.165	1.571(0.703, 3.509)	0.270
38–47	0.769(0.464, 1.273)	0.307	1.294(0.673, 2.490)	0.439
48–57	0.381(0.218, 0.668)	0.001	1.343(0.750, 2.403)	0.321
58 and above	0.365(0.200, 0.666)	0.001	0.961(0.525, 1.762)	0.899
Sex				
Female	1.000		1.000	
Male	1.236(0.957, 1.597)	0.104	1.198(0.828, 1.734)	0.338
Marital status				
Else	1.00		1.000	
Married	1.178(0.828, 1.675)	0.362	1.555(0.998, 2.422)	0.051
Number of children				
1 or 2	1.000		1.000	
3–4	1.101(0.815, 1.487)	0.532	1.018 (0.599, 1.732)	0.946
Other	1.069(0.727, 1.571)	0.736	1.130 (0.669, 1.910)	0.648
Location of respondent				
Ipokia LGA	1.000		1.000	
Yewa North LGA	0.480(0.350, 0.657)	0.001	2.030(1.233, 3.345)	0.005
Imeko-Afon LGA	0.589(0.408, 0.849)	0.005	0.524(0.331, 0.831)	0.006
Duration in location (Years)				
10 or less	1.000		1.000	
11–30	2.258(1.350, 3.778)	0.002	0.431(0.207, 0.899)	0.025
31 or more	2.323(1.329, 4.060)	0.003	0.590(0.364, 0.958)	0.033
Level of education				
No formal education/ Primary education	1.000		1.000	
Secondary/ Post Secondary education	0.978(0.751, 1.275)	0.872	1.409(0.715, 2.776)	0.322
Tertiary education	0.586(0.346, 0.993)	0.047	1.648(0.870, 3.122)	0.125
Income level				
Less than N30,000	1.000		1.00	
N30,000–N69,999	1.211(0.901, 1.628)	0.205	0.338(0.206, 0.553)	0.001
N70,000 or more	1.152(0.799, 1.661)	0.448	0.597(0.374, 0.952)	0.030
Occupation				
Not working	1.000		1.000	
Self-employed	1.428(0.778, 2.620)	0.250	1.673(0.657, 4.259)	0.281
Farming	1.277(0.664, 2.456)	0.464	1.231(0.557, 2.718)	0.607
Trading	0.885(0.491, 1.597)	0.685	0.800(0.326, 1.963)	0.626
Artisan	1.144(0.628, 2.084)	0.661	0.629(0.293, 1.352)	0.235
Civil Servant/ Other	2.268(1.158, 4.445)	0.017	0.530(0.231, 1.221)	0.136
Religion				
Traditional/ Other	1.000		1.000	
Islam	1.107(0.625, 1.962)	0.727	0.719(0.337, 1.535)	0.394
Christian	0.933(0.529, 1.643)	0.809	0.769(0.533, 1.111)	0.162

Socio-demographic factors	Model 3		Model 4		Model 5	
	Care through hospital treatment		Care through traditional medicine		Care through self-medication	
	OR (95% C. I)	Sig.	OR (95% C. I)	Sig.	OR (95% C. I)	Sig.
Age (Years)						
18–27	1.000		1.000		1.000	
28–37	0.793(0.400, 1.573)	0.507	4.245(1.811, 9.950)	0.001	1.343(0.661, 2.728)	0.414
38–47	0.961(0.555, 1.665)	0.888	1.606(0.830, 3.108)	0.160	0.815(0.461, 1.443)	0.483
48–57	0.970(0.592, 1.592)	0.905	1.405(0.776, 2.543)	0.262	0.922(0.552, 1.538)	0.755
58 and above	1.051(0.638, 1.730)	0.845	1.639(0.875, 3.071)	0.123	0.858(0.511, 1.441)	0.563
Sex						
Female	1.000		1.000		1.000	
Male	1.221(0.901, 1.655)	0.198	1.068(0.729, 1.564)	0.735	0.834(0.605, 1.148)	0.264
Marital status						
Else	1.000		1.000		1.000	
Married	0.606(0.415, 0.884)	0.009	1.685(1.039, 2.732)	0.034	1.161(0.781, 1.724)	0.460
Number of children						
1 or 2	1.000		1.000		1.000	
3–4	0.926(0.596, 1.438)	0.732	1.353(0.786, 2.328)	0.276	1.232(0.779, 1.949)	0.372
Other	0.857(0.555, 1.324)	0.488	1.136(0.669, 1.929)	0.638	1.539(0.976, 2.428)	0.063
Location of respondent						
Ipokia LGA	1.000		1.000		1.000	
Yewa North LGA	0.629(0.399, 0.991)	0.046	0.951(0.586, 1.544)	0.839	1.224(0.768, 1.950)	0.372
Imeko-Afon LGA	0.752(0.512, 1.105)	0.146	5.120(3.137, 8.356)	0.000	3.581(2.406, 5.331)	0.001
Duration in location (Years)						
10 or less	1.000		1.000		1.000	
11–30	1.304(0.705, 2.412)	0.398	0.155(0.078, 0.310)	0.001	0.900(0.478, 1.695)	0.745
31 or more	1.503(1.005, 2.247)	0.047	0.471(0.295, 0.753)	0.002	0.898(0.592, 1.364)	0.615
Level of education						
No formal education/ Primary education	1.000		1.000		1.000	
Secondary/ Post Secondary education	0.764(0.436, 1.339)	0.347	1.801(0.928, 3.496)	0.082	0.972(0.545, 1.735)	0.925
Tertiary education	0.844(0.496, 1.438)	0.533	1.337(0.719, 2.486)	0.359	0.830(0.479, 1.438)	0.506
Income level						
Less than N30,000	1.000		1.000		1.000	
N30,000–N69,999	1.270(0.829, 1.947)	0.272	1.306(0.778, 2.192)	0.313	1.375(0.881, 2.145)	0.161
N70,000 or more	0.868(0.567, 1.331)	0.517	1.532(0.921, 2.549)	0.101	0.932(0.599, 1.450)	0.754
Occupation						
Not working	1.000		1.000		1.000	
Self-employed	0.661(0.288, 1.520)	0.330	1.103(0.400, 3.040)	0.849	0.775(0.328, 1.835)	0.563
Farming	1.317(0.651, 2.662)	0.443	0.778(0.334, 1.813)	0.561	0.436(0.209, 0.910)	0.027
Trading	1.080(0.515, 2.268)	0.838	1.149(0.442, 2.988)	0.776	0.722(0.331, 1.573)	0.412
Artisan	0.809(0.422, 1.549)	0.522	1.337(0.586, 3.049)	0.490	0.789(0.399, 1.563)	0.497
Civil Servant/ Other	0.577(0.288, 1.156)	0.121	1.325(0.548, 3.206)	0.532	1.278(0.612, 2.668)	0.513
Religion						
Traditional/ Other	1.000		1.000		1.000	
Islam	0.557(0.284, 1.091)	0.088	0.755(0.351, 1.628)	0.474	0.937(0.473, 1.856)	0.851
Christian	0.903(0.673, 1.212)	0.498	0.818(0.560, 1.196)	0.301	0.801(0.587, 1.092)	0.160

Level of Significance; *p* ≤ 0.05. Model 1: dependent variable = sick in the last 12 months; *N* = 1,317. Model 2: dependent variable = frequency of sickness; *N* = 855. Model 3: dependent variable = care through hospital treatment; *N* = 855. Model 4: dependent variable = care through traditional medicine; *N* = 855. Model 5: dependent variable = care through self-medication; *N* = 855.

Model 1 shows the odds of respondents reporting any sickness in the last 12 months before the study. The results show that the odds that respondents became sick in the last 12 months was lower for those aged 48–57 years (OR = 0.381; CI = 0.218, 0.668), and 58 years and above (OR = 0.365; CI = 0.200, 0.666) compared to the reference age group (18–27 years). Also, the odds that the respondents reported sick in the last 12 months was lower for residents of Yewa North LGA (OR = 0.480; CI = 0.350, 0.657), and Imeko-Afon LGA (OR = 0.589; CI = 0.408, 0.849), compared to the reference location (Ipokia LGA). In addition, the odds that the respondents had sickness in the last 12 months was higher for those who have lived in the border communities for 11–30 years (OR = 2.258; CI = 1.350, 3.778), and 31 years or more (OR = 2.323; CI = 1.329, 4.060), compared to the reference category (10 or less years). Moreover, the odds that the respondents felt sick in the last 12 months was lower for the residents who were tertiary educated (OR = 0.586; CI = 0.346, 0.993), compared to the reference category (no formal education/primary education).

Model 2 shows the odds of frequency of sickness for respondents who stated that they had being sick within the last 12 months. The results indicate that the odds of frequent sickness was higher for respondents who live in Yewa North LGA (OR = 2.030; CI = 1.233, 3.345), and lower for people who reside in Imeko-Afon LGA (OR = 0.524; CI = 0.331, 0.831), compared to the reference category (Ipokia LGA). Also, the odds of becoming sick on regular basis was lower for respondents who earn 30,000 naira to 69,999 naira per month (OR = 0.338; CI = 0.206, 0.553), and 70,000 naira or more per month (OR = 0.597; CI = 0.374, 0.952), compared to the reference category (less than 30,000 naira per month).

Model 3 signifies the odds of seeking care through hospital treatment for respondents who became sick within the last 12 months. The odds of getting care through hospital treatment was lower for residents who were married (OR = 0.606; CI = 0.415, 0.884), compared to the reference category (else). Similarly, the odds of seeking care through hospital treatment was lower for respondents who live in Yewa North LGA (OR = 0.629; CI = 0.399, 0.991), compared to the reference category (Ipokia LGA). Also, the odds of seeking care through hospital treatment was higher for residents who have stayed in these communities for 31 or more years (OR = 1.503; CI = 1.005, 2.247), compared to the reference category (10 or less years).

Moreover, Model 4 indicates the odds of seeking care through traditional medicine for respondents who reported being sick within the last 12 months. The odds of seeking care through traditional medicine was higher for respondents aged 28–37 years (OR = 4.245; CI = 1.811, 9.950), compared to the reference category (18–27 years). Similarly, the odds of procuring care through traditional medicine was higher for respondents who were married (OR = 1.685; CI = 1.039, 2.732), compared to the reference category (else). Also, the odds of seeking care through traditional medicine was higher for respondents who live in Imeko-Afon LGA (OR = 5.120; CI = 3.137, 8.356), compared to the reference category (Ipokia LGA). In addition, the odds of assessing care through traditional medicine was lower for residents who have lived in these communities for 11–30 years (OR = 0.155; CI = 0.078, 0.310), and 31 or more years (OR = 0.471; CI = 0.295, 0.753), compared to the reference category (10 or less years).

Furthermore, Model 5 shows the odds of getting care through self-medication for respondents who stated that they were sick during the last 12 months before the study. The odds of seeking care through self-medication was higher for residents who live in Imeko-Afon LGA (OR = 3.581; CI = 2.406, 5.331), compared to the reference category (Ipokia LGA). Also, the odds of self-medicating was lower for respondents who were farmers (OR = 0.436; CI = 0.209, 0.910), in comparison with the reference category (not working).

## 4 Discussion

The findings of this study reveal salient relationships that exist between respondents’ socio-demographic factors, health conditions and treatment-seeking choices within these border communities. The results support and amplify existing literature, while offering valid contributions on the connections between socio-demographic factors, health conditions and treatment-seeking choices among individuals within these border communities. Table 1 shows that about 56.2% of the respondents are aged 28 to 47 years. This statistic represents the core of the prime age working population, who are majorly the drivers of economic activities, formation of families, and public consumption. This suggests the need for adequate and targeted economic empowerment of these residents, so as to rejig their wealth potential and capacity, leading to economic prosperity in these localities. Also, 74.2% of the residents are married, highlighting the necessity for investments in reproductive health matters such as family planning, infant and maternal health. In addition, only about 10.8% of the respondents have more than secondary education, showcasing the dire need to improve the educational capacities of these residents, by adequate investment in educational institutions, and subsidized or free schooling, which will in turn enhance their health behavior and choices.

In Table 2, results in Model 1 show that respondents aged 18 to 27 years are more likely to feel sick in these communities. Younger people who are mostly active may report more transient sicknesses (such as head-ache or body pains) which may easily be captured by a simple ‘sick in the last 12 months’ binary measure, compared to older individuals. Pagan (26) argued that young people aged 18 to 26 are still undergoing emotional, social and cognitive development, making it a susceptible time for health challenges, as they encounter new stressors. However, since this study relies on unverified self-reported health status; the perception of being ill is likely to vary with age, and may require longitudinal studies or medical records to confirm this trend. Also, the results in Model 1 indicate that people who live in Ipokia LGA are more prone to sicknesses than in the other two LGAs. Idi-iroko community, in Ipokia LGA is where the official border gateway between Nigeria and the Benin Republic (through Ogun State) is located. The higher rate of cross-border related activities such as smuggling of illegal goods, stringent border security methods and infiltration of unchecked migrants could likely contribute in the greater prevalence of sicknesses in this LGA.

In addition, findings in Model 1 show that people who have stayed for more than 10 years in the border communities were more susceptible to sicknesses compared to those that have lived for 10 years or less. The reason for this result may be because residents who have lived in these border communities for longer periods have become entrenched in socio-economic patterns associated with poor health outcomes. Hence these set of people might have been neglected for a longer time, and lacked adequate access to basic amenities such as clean drinking water and adequate healthcare access, necessary for healthy living. Also, due to their longer existence in these border areas, they may be more involved in illegal cross-border related socio-economic activities such as smuggling of goods, with its attendant negative health consequences. Also, the statistics in Model 1 indicate that residents who are tertiary educated are less likely to feel sick in these communities. This implies that education raises the awareness of people to make better health choices, which in turn influences their health outcomes positively. Nachinaab et al. (16) reiterated that there was significant association between patients’ level of education and their health-seeking behavior with respect to the utilization of private and public health facilities. Hence it is imperative that the government increase investments in education in these localities by building and equipping more schools, and subsidizing fee payments through scholarships and/or free education, in order to encourage the residents to acquire necessary knowledge that will inevitably improve their health behavior.

In Model 2, the results show that people who live in Yewa North are more likely to fall sick compared to residents of Ipokia LGA and Imeko-Afon LGA. This could be because of the considerable larger population size of Yewa North LGA and apparent lack of necessary health facilities and affordable healthcare services to properly cover and cater to the health of these residents, compared to the lower population sizes of Ipokia LGA and Imeko-Afon LGA. Similarly, results in Model 2 show that earning higher income reduces the exposure of these residents to recurrent sicknesses. This implies that the more income the residents earn, the more they have the financial capacity to properly take care of their health. Claims by Armenti, Sweeney, Lingwall & Yang (11) who asserted that individuals with greater economic power and resources are more likely to be healthier than people in deprived positions support this result. Also, Chukwu et al. (19, 20) stated that border residents with low economic status usually experience limited access to healthcare services. This calls for adequate policy interventions to boost the income opportunities of these residents, which will in turn enhance their health outcomes. Hence it is advisable that the government provide vocational training centers, microcredit programs and easy access to cross-border trade, among others, in order to enhance the income opportunities of these residents, which will inadvertently improve their health outcomes.

In addition, results in Model 3 signify that respondents who are married are less likely to seek care in hospitals. This is supported by Nachinaab et al. (16), who reiterated that the marital status of individuals may influence their health-seeking behavior with respect to the utilization of private and public health facilities. More than two-thirds (71.8%) of married women in Ethiopia (with most residing in rural areas) reported barriers to accessing health care services, as a result of low income capacity (27) It is possible that the high cost of healthcare services for the residents in these communities may likely dissuade married people (who may be struggling with other family expenses) from seeking care in the hospitals. Chukwu et al. (19, 20) implied that border residents with low economic wherewithal usually experience limited access to health services. Results in Table 1 show that majority of the respondents are low income earners. It is possible that the relative high cost of hospital healthcare services in these communities may influence residents (such as married people- who may likely have more financial responsibilities) to seek care through other possible cheaper alternatives, instead of hospital care. This may be because they also want to meet up with other pressing family needs. According to Fantin et al. (10), individuals with low economic status usually experience significant difficulties accessing basic healthcare services. It is also likely that the inadequacy of health services in these communities, perhaps concerning reproductive health matters, may discourage married residents from visiting the hospitals for care. This is supported by Oladosun et al. (23) who emphasized that rural communities in Nigeria lack adequate health facilities and personnel. Hence, it is imperative that the government provide adequate health facilities and services, and also reduce or subsidize the cost of hospital healthcare services in these border areas, in order to encourage more residents to seek proper medical care from trained professionals in hospitals, thereby positively influencing the health outcomes of these border settlements.

Similarly, results in Model 3 indicate that residents of Yewa North LGA are less likely to seek care through hospital treatment, compared to those in Ipokia LGA. The comparable larger population size of Yewa North LGA and apparent lack of enough health facilities to adequately cater to the people’s healthcare needs could be the reason for this statistic. An individual’s area of residence may influence access to public services (9). Similarly, the results show that residents who have stayed longer in these communities are more likely to seek cure through hospital treatment. It is possible that residents, who have lived in these communities for longer years, have a better established hospital healthcare seeking access, (especially across the border) than people who just came into the community.

Moreover, findings in Model 4 reveal that residents aged 28–37 years are more likely to seek care through traditional medicine. Residents aged 28–37 years may likely have more financial responsibilities to attend to, hence prompting them to seek care through traditional medicine, which may be cheaper (so as to meet up with other pressing needs), compared to people aged 18–27 years, and with less financial responsibilities. Also, the results show that respondents who are married are more likely to seek care through traditional medicine. The apparent high cost of procuring healthcare in the hospitals by the residents, as a result of their low economic status could prompt people who are married to seek care through traditional medicine which is likely cheaper and affordable, in order to meet up with other family expenses. In addition, the results indicate that respondents in Imeko-Afon LGA are more likely to seek care through traditional medicine, compared to people in Ipokia LGA. It is likely that residents of Imeko-Afon LGA do not have access to hospital healthcare services as much as people in Ipokia LGA, influencing them to seek care through other means such as traditional medicine. According to The Lancet (9), people’s place of residence may shape their access to public goods and services. The results also show that people who have lived in these communities for more number of years are less likely to seek care through traditional medicine. As indicated previously, there is a possibility that residents who have lived in these localities for higher number of years, have a more established hospital healthcare seeking access, compared to new entrants.

Furthermore, results in Model 5 indicate that residents of Imeko-Afon LGA are more likely to self-medicate, compared to people in Ipokia LGA. Residents of Imeko-Afon LGA are apparently more likely to seek care through self-medication, perhaps due to lack of access to hospital health services, as compared to Ipokia LGA. Hence, the government should provide more healthcare facilities and personnel in these border areas, so as to enhance their access to health services, which will also improve well-being in these localities. Also, the result show that respondents who are farmers are less likely to self-medicate compared to people who are not working. People who are farmers are likely to earn more income, and be able to afford the cost of healthcare services in hospitals than residents who are not working. Hence, farmers may be more inclined to get care from health professionals in the hospitals, instead of self-medicating, in comparison to those who are not working.

### 4.1 Limitations of the study

The cross-sectional design of the study did not permit causal inferences to be drawn, only associations were found. Besides, health status as reported by the respective respondents is subjective, as there were no clinical tests to ascertain the respondents’ claims. There was also the possibility of recall bias, whereby the respondent does not fully remember the details of past events, due to time past. Also, the study was limited to selected border LGAs in Ogun State Nigeria which are mainly rural areas, and hence findings may not be generalizable to other settings. Moreover, the study did not ask the respondents whether they sought cure across the border, which is common in border areas.

## 5 Conclusion

From the results, it can be observed that socio-demographic factors show relevant association with the health conditions of residents in border communities of Ogun State Nigeria, and also reveal salient relationships among the variables tested, highlighting the vulnerabilities associated with living in border areas. This study is relevant as it may inform efforts to promote healthy living in border communities in Nigeria, and also assist in the attainment of Sustainable Development Goal 3 (SDG3) in the nation. Hence the study recommends the following.

1) The government should provide more healthcare facilities and personnel in these border areas, so as to enhance their access to health services, which will also improve well-being in these localities.
2) The government should reduce or subsidize the cost of hospital healthcare services in these border areas, in order to encourage more residents to seek proper medical care from trained professionals in hospitals, thereby influencing the health outcomes of these communities positively
3) The government should improve the economic status of these border areas by providing economic empowerment and employments to the residents, as this will help the residents to take proper care of their health.
4) The government should increase investments in education in these communities by building more schools, offering free education and scholarships to these residents, so as to improve their level of education, which will inadvertently improve their health outcomes.

## Data Availability

The raw data supporting the conclusions of this article will be made available by the authors, without undue reservation.
